# Ventilation inhomogeneities in children with congenital thoracic malformations

**DOI:** 10.1186/s12890-015-0023-1

**Published:** 2015-03-25

**Authors:** Payal H Mandaliya, Matthew Morten, Rajendra Kumar, Alan James, Aniruddh Deshpande, Vanessa E Murphy, Peter G Gibson, Bruce Whitehead, Paul Robinson, Joerg Mattes

**Affiliations:** Experimental & Translational Respiratory Medicine, Hunter Medical Research Institute, University of Newcastle, Newcastle, NSW Australia; Paediatric Respiratory and Sleep Medicine Department, Newcastle Children’s Hospital, Newcastle, NSW Australia; Paediatric Surgery Department, Newcastle Children’s Hospital, Newcastle, NSW Australia; Hunter Medical Research Institute, University of Newcastle, Newcastle, NSW Australia; Surgery Department, John Hunter Hospital, Newcastle, NSW Australia; Paediatric Respiratory Department, Children’s Hospital at Westmead, Sydney, NSW Australia

**Keywords:** Lung inhomogeneities, Lung clearance index (LCI), Nitrogen multiple breath washout, Reactance, Resistance, Lung function, Congenital thoracic malformations

## Abstract

**Background:**

Congenital thoracic malformations (CTM) are rare lung lesions that are managed with surgical resection or active surveillance.

The objective of this study was to comprehensively assess large and small airway function in children with CTM who underwent lobectomy in early life.

We hypothesise that sensitive measures of lung function will demonstrate residual impairments in CTM compared to healthy children.

**Methods:**

Nitrogen lung clearance index (LCI), reactance and resistance (X5Hz and R5Hz), forced expiratory volume in 1 s and forced vital capacity (FEV1 and FVC) were prospectively measured in 10 children with CTM (mean age/SD: 7.6/1.3) who had undergone surgical resection in early life and in 17 healthy children (mean age/SD: 4.8/0.4). Total lung capacity (TLC) was also conducted in children older than 7 years of age with CTM (n = 8).

**Results:**

Mean LCI was 8.0 (95% CI 7.5 to 8.5) in the CTM group and 7.3 (95% CI 7.0 to 7.6) in healthy children (p = 0.016). Mean X5Hz was −0.44kPa/l/s (95% CI −0.58 to −0.31) in the CTM group and −0.31kPa/l/s (95% CI −0.35 to −0.27) in healthy children (p = 0.02). Mean Z score for X5Hz was −2.11 (95% CI −3.59 to −0.63) in the CTM group and −0.11 (95% CI −0.55 to 0.33) in healthy children (p = 0.0008). Mean FEV1 was 1.21 L (95% CI 0.97 to 1.45) in the CTM group and 1.02 L (95% CI 0.90 to 1.15) in healthy children (p = 0.22). Mean % predicted FEV1 was 83% (95% CI 74 to 92) in the CTM group and 97% (95% CI 87 to 107) in healthy children (p < 0.05). Mean % predicted TLC in CTM children was 121.3% (95% CI 88.45 to 154.1). Mean LCI was inversely correlated with height z-scores in the CTM group (rs = −0.88, p = 0.002) but not in healthy children (rs = 0.22, p = 0.4).

**Conclusions:**

Children with CTM have impaired lung function as demonstrated by the significant differences in LCI, reactance and FEV1 but not FVC, resistance and TLC. These findings may be of clinical relevance as ventilation inhomogeneities are closely correlated with somatic growth in this study.

**Electronic supplementary material:**

The online version of this article (doi:10.1186/s12890-015-0023-1) contains supplementary material, which is available to authorized users.

## Background

Congenital cystic adenomatoid malformations (CCAM) are rare with an incidence of about one case per 10,000 to 35,000 births [1]. CCAMs have been classified traditionally by histological classification given by Stocker with three types (I, II and III) and the addition of type 0 and type IV subsequently [2]. CCAM type I are macrocystic, type II are microcystic and often associated with multiple congenital anomalies, type III are solid, type 0 have bilateral involvement and type IV have an association with pleuropulmonary blastoma. A new terminology for CCAM was subsequently also suggested by Stocker (congenital pulmonary airway malformation (CPAM)) [3]. Most recently the term congenital thoracic malformations (CTM) was coined by Bush [4] to include a spectrum of lung lesions that are macroscopically often indistinguishable, namely CCAM, sequestration, congenital lobar emphysema, enteric and bronchogenic cysts, and bronchial atresia but excludes congenital diaphragmatic hernia.

The prognosis of CTM is highly variable as determined by multiple factors including size of lung mass, presence of lung hypoplasia, polyhydramnios, mediastinal shift, and fetal hydrops. While many of the cystic lung lesions regress in-utero, some fail to do so. The cystic lung lesions have been controversial in terms of nomenclature, mechanism of pathogenesis and classification of lesions. In regards to approaches to management there exists consensus regarding surgical intervention in cases with fetal hydrops and in symptomatic newborns with CTM.

In non-operated cases, there is a risk of sudden enlargement of cysts, infections, pneumothorax and malignancy. In particular CCAM type II is associated with the development of pleuropulmonary blastoma, while rhabdomyosarcoma, myxosarcoma, and bronchioloalveolar carcinoma have been observed in CCAM tissue [4]. Early surgery for CTM is recommended by some authors given the above risks. However malignant transformation remains uncommon and may occur months after surgical resection [5-7]. Consensus is also lacking regarding appropriate monitoring for malignancies in non-resected CTM subjects.

Notably the natural history of CTM is not very well documented, and hence it is difficult to determine its most appropriate management. In fact data on long-term respiratory outcomes in children with CTM is scarce. Dysanaptic growth of the remaining lung tissue has been described following resection of congenital lung lesions in humans and post-pneumonectomy in animals by mechanisms of alveolar distention and neovascularization with new alveolar formation while the conducting (small/peripherial) airways do not participate equally [8,9]. The potential consequences include increase in airways resistance, change in elastic recoil properties of the lung tissue, and the occurrence of ventilation inhomgeneities. However, normal lung function, as measured by spirometry, has been reported in children with CTM resected in early life [10].

The peripheral, or small airways defined as conducting airways that are less than 2 mm in internal diameter and extend from the noncartilaginous bronchioles to the alveolar ducts are anatomically connected to the regenerating alveolar compartment, hence abnormalities in lung function associated with resected CTM is more likely to represent peripheral airway abnormalities. The peripheral airways are the predominant site of gas mixing within the lungs, and account for 95% of the total lung volume [11]. However, these airways are largely ignored by spirometry, which measure bulk airflow and predominantly reflect more central airway function [12]. As a result spirometry indices such as FEV_1_ can remain normal despite marked involvement of the peripheral airways in disease processes.

Recently, other physiological measures of lung function, possibly more sensitive, have become available and are validated in children. Multiple breath washout (MBW) is a non-invasive tidal breathing test, which assesses the gas mixing efficiency within the lungs [13], several studies have confirmed the superior sensitivity of MBW tests to detect these changes in important paediatric respiratory diseases [11]. Impulse oscillometry (IOS) evaluates the mechanical properties of the respiratory system during tidal breathing. Airway diameter with changes in lung volume, or so-called airway distensibility is reflected by reactance, and changes in airway pressure with lung volume by resistance. IOS has been demonstrated to detect small changes in pulmonary function in children with mild asthma and eosinophilic bronchitis, which were undetected by spirometry [14]. The lung growth post resection of CTM in humans may not be entirely normal and the dysanaptic growth may be reflected by changes in airway mechanics.

MBW and IOS are emerging as powerful tools in the early detection of pulmonary function in children unable to perform a maximal forced expiration [15,16]. Based on these findings, we have employed nitrogen MBW and IOS to determine if following lobectomy, residual abnormalities are present at school age in children with CTM compared to healthy controls.

## Methods

### Population

The study involved 10 children recruited from our surgical database who had undergone surgery for suspected CTM in early life at the Newcastle Children’s Hospital and 17 healthy children without a diagnosis of asthma, current or previous CTM or any chronic respiratory symptoms or disease. A diagnosis of “asthma ever” was assessed in a standardised questionnaire [17] and a diagnosis of “doctor’s diagnosis of asthma” by a standardised parent interview. The study was approved by the Hunter New England Research Ethics Committee and written informed consent was obtained from parents or legal guardians before inclusion into the study.

### Study design

Participants performed lung function testing on the same day beginning with nitrogen MBW, followed by IOS and spirometry. Skin prick testing to common inhaled allergens was also performed to assess the atopic nature of participants. In those children old enough we preformed lung volumes (>7 years).

### Measurement of lung function

*Nitrogen MBW* was performed using recently validated commercial MBW (Exhalyzer D, EcoMedics AG, Switzerland), which is an open circuit, bias flow system along with associated software (Spiroware 3.1 EcoMedics AG, Switzerland). N_2_ concentration is calculated indirectly by measuring O_2_ and CO_2_ concentration (Capnostat 5, Philips Healthcare, The Netherlands) simultaneously. The participant breathed in 100% oxygen during washout until N_2_ concentration was reduced to at least 1/40 th of the initial concentration [18]. During testing the patient was closely monitored for any signs of leak. The test was performed in a seated position whilst watching a movie to encourage stable tidal breathing. The switch from room air to 100% oxygen was manually activated following an acceptable stable tidal breathing pattern and end-expiratory lung volume, in accordance with recent consensus. LCI was reported as the mean of at least two acceptable tests within 10% of each other [18].

*IOS measurements* (MasterScreen system, Jaeger Co, Wuerzberg, Germany) were made according to ERS task force recommendations [19]. The child was asked to breathe in a relaxed manner whilst seated in an upright position with a nose clip in place, while both cheeks and the tongue base were supported with two hands by the operator. Mean reactance and resistance were calculated over a measurement period of 30 seconds using CareFusion software (CareFusion, Australia) at frequencies of 5 Hz (R5Hz and X5Hz, respectively). The predicted value of at least two reproducible replicates (coefficient of variation of at least 2 sets of data < 10%) [20] were reported using published reference data [21].

*Spirometry* (MasterScreen system, Jaeger Co, Wuerzberg, Germany) was conducted in accordance to ATS/ERS guidelines [22] using the global lung initiative (GLI) of 2012 [23].

### Skin prick allergy test

Skin prick testing was conducted using standard extract for environmental allergens (house dust mites, cat and dog dander, rye grass, 12 grass mix), 1:10 w/v (Stallergènes®, Antony, France). A positive result was defined as 3 mm or greater than negative saline control, determined by averaging maximal perpendicular wheal diameters 15 minutes after applying the lancet. Positive control was with histamine base, 6 mg/ml (Stallergènes®, Antony, France) and had to be 3 mm or greater for a valid test. Participants were asked to withhold any antihistamines for three days prior to the test.

### Statistical analysis

Statistical significance was determined using a nonparametric, two-tailed Mann–Whitney U-test or the parametric t-test as appropriate. Correlation analyses were performed using a nonparametric Spearman’s test. Age-adjusted height and weight z-scores were calculated using the 2000 Centres for Disease Control and Prevention Growth Charts [24]. We aimed for a participation of at least 9 children per group to detect a mean difference of 1.5 SD in LCI (80% power, α = 5%) between the groups, which we defined as clinically relevant.

## Results

17 children with CTM were identified in our medical record database but 5 families were not contactable. 10 of the 12 contacted parents agreed to participate (participation rate 83%) while one family declined and one child lived too distant to attend. All children performed spirometry and MBW while IOS were unsuccessful in 2 patients due to instrument error. Results are compared to data from 17 healthy children participating in an ongoing longitudinal study [25]. MBW and spirometry was successful in 16 and IOS in 17 healthy children. The age range in healthy children was 4 to 5 years and in children from the CTM group 5 to 9 years (Table 1). Thus children from the control group were slightly lighter and shorter, however did not differ in their age-adjusted weight and height Z-scores from children with CTM (Table 1). Four of 10 children from the CTM group had a doctor’s diagnosis of asthma and had documented salbutamol use, but none in the group of healthy children as per inclusion criteria (Table 1). As expected, more CTM children had wheeze ever (70%) when compared to healthy children (24%; p = 0.04) while there was no significant difference in the prevalence of other respiratory symptoms (Additional file 1: Table S1).

**Table 1:** **Demographic details**

	**CTM N (%) OR mean (SD)**	**Controls N (%) OR mean (SD)**	**P value**
Total	10	17	
Age	7.6 (1.3)	4.8 (0.4)	<0.01
Male	4 (40%)	4 (24%)	0.41
Mean weight (kg)	24.9 (6.3)	18.7 kg (2.1)	ND
Mean height (cm)	123.4 (10.1)	108.6 (4.0)	ND
Mean Z score weight	−0.2 (1.2)	0.4 (1.0)	0.16
Mean Z score height	−0.3 (1.9)	0.5 (0.9)	0.06
Prematurity (<37 completed weeks)	3 (30%)	1 (6%)	0.13
Bronchiolitis in infancy	2 (20%)	2 (12%)	1.0
Asthma	4 (40%)	None as per inclusion criteria	NA
Eczema	4 (40%)	3 (18%)	1.0
Allergic rhinitis	1 (10%)	2 (12%)	0.56
Positive skin prick test	1 (10%)	1 (6%)	1.0

Not applicable (NA), not determined (ND).

**Figure 1:**
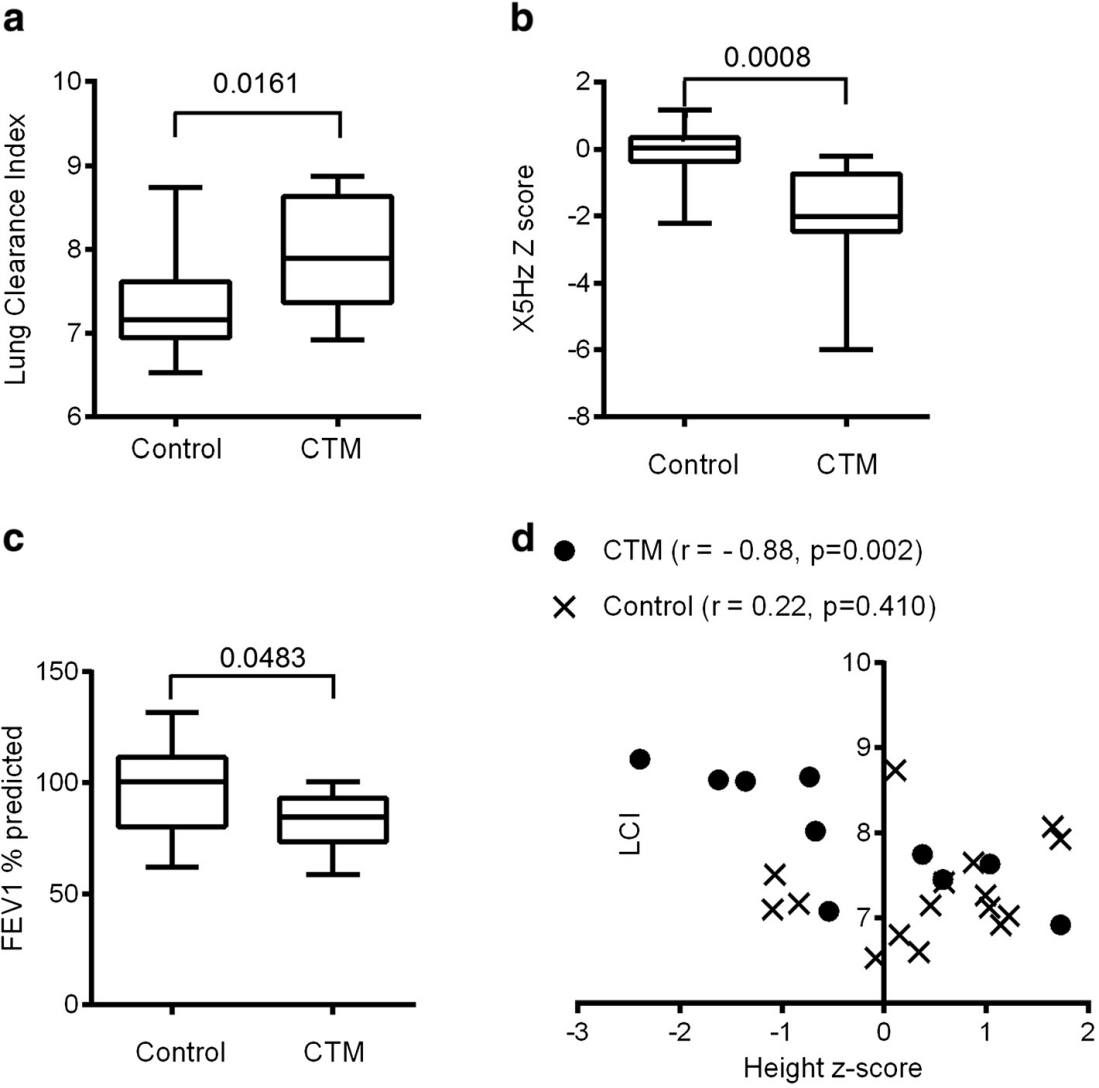
**Lung function results of children with CTM and healthy controls: a) LCI, b) reactance Z scores, c) FEV**_**1**_ **% predicted, d) correlation between LCI and age-adjusted height Z scores in children with CTM compared to healthy children.**

**Figure 2:**
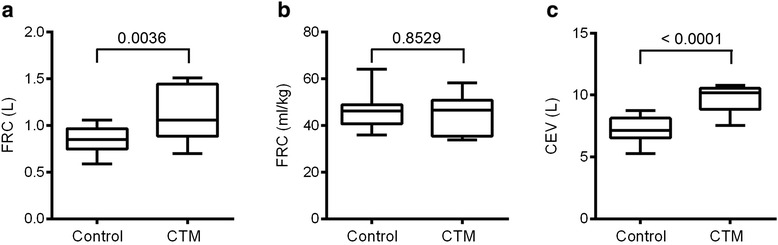
**N**_**2**_ **MBW lung volumes in children with CTM and healthy controls: a) FRC (L), b)FRC (ml/kg), c) CEV (L).**

When comparing lung function values, children from the CTM group had a significantly higher lung clearance index (LCI), and lower FEV_1_% predicted and reactance (X5Hz) Z score values (Table 2 and Figure 1a to c). However airway resistance (R5Hz), FVC% predicted and FEV_1_/FVC Z-scores were not different to the values measured in the healthy control group (Table 2). The CTM group showed lower functional residual capacity (FRC) values (Figure 2a) as they were younger but this was not evident if FRC was corrected for weight (Figure 2b). The cumulative expired volume (CEV) was higher in the CTM group (Figure 2c).

**Table 2:** **Lung function test results**

	**CTM group Mean/SD**	**(n)**	**Control group Mean/SD**	**(n)**	**P value**
**Multi breath washout**					
**Mean LCI**	**8.0/0.7 (10)**	**10**	**7.3/0.6**	**16**	**0.02**
**Mean FRC**	**1.11**	**10**	**0.85**	**16**	**<0.01**
**Spirometry**					
**Mean FEV**_**1**_ **(%predicted)**	**83.0/12.7**	**10**	**97.0/18.7**	**16**	**<0.05**
Mean FVC (%predicted)	94.2/16.2	10	102.7/21.3	16	0.29
Mean FEV_1_/FVC (%predicted)	88.4/11.0	10	94.7/7.6	16	0.09
Mean TLC (%predicted)	121.3/39.2	8			
**Impulse Oscillometry**					
**Mean X5Hz (Z score)**	**−2.11/1.768**	**8**	**−0.11/0.86**	**17**	**<0.01**
Mean R5Hz (Z score)	1.45/1.77	8	0.51/1.15	17	0.12

The details of pulmonary function tests of the CTM group along with histopathological diagnosis and somatic growth parameters are given in Table 3. The details of pulmonary function tests of the control group are given in Additional file 1: Table S2. The mean % predicted total lung capacity (TLC) of children with CTM was 121.3% (95% CI 88.45 to 154.1). If only non-asthmatic CTM children were included in the analysis there remained a significant difference in LCI (difference 0.7; 95% CI 0.2 to 1.3, p = 0.01) and X5 (difference in % predicted: 61.2%; 95% CI 31.7 to 90.7, p = 0.0003) but not FEV_1_% (difference in % predicted: 7.1%; 95% CI −9.6 to 23.8, p = 0.39) as compared to healthy children. Interestingly a significant inverse correlation between age-adjusted height Z-scores and LCI was observed in children from the CTM cohort but not in healthy children (Figure 1d). This correlation remained even when the outlier was excluded (data not shown). This was neither the case for weight Z scores nor weight and height not adjusted for age or any other lung function parameters (data not shown).

**Table 3:** **Details on histopathology and lung function in children with CTM and resection in early life**

**Age/sex**	**Asthma**	**Site**	**Pathology**	**Surgery (age in months)**	**Height Z score**	**LCI**	**FRC (L)**	**FEV**_**1**_ **% pred**	**FVC % pred**	**TLC % pred**	**R5Hz Zscore**	**X5Hz Zscore**
9.5/F	Y	LUL	Bronchial atresia + CCAM	36	1.7	6.9	1.46	88	91	211	X	X
7.3/F	N	RLL	CCAM Type III	2.5	−1.4	8.6	0.93	92	89	110	−0.18	−0.21
9.0/F	N	LUL	CCAM Type II/III	9	−2.4	8.9	1.06	90	125	128	1.34	−1.91
6.2/M	N	LLL	Intralobar sequestration	15	−0.7	8.7	0.73	81	85	X	1.53	−2.52
7.4/M	N	RLL	Congenital sequestration	9	−0.7	8.0	1.04	82	95	77	0.82	−2.26
6.9/M	N	LUL	Mixed type CCAM	0.5	1.0	7.6	1.20	100	113	108	X	X
8.2/F	Y	RLL	CCAM type IV	0.3	−1.6	8.6	1.06	59	74	119	5.05	−5.98
9.0/F	N	LLL	Intralobar sequestration	9.5	−0.5	7.1	1.44	95	104	102	2.55	−2.13
7.4/M	Y	RUL	Unclassified CTM	13	0.4	7.8	1.51	69	94	115	-.067	−0.52
5.7/F	Y	RLL	CCAM type II/III	0.3	0.6	7.5	0.70	75	73	X	1.17	−1.38

Left upper lobectomy (LUL), right lower lobectomy (RLL), left lower lobectomy (LLL), right upper lobectomy (RUL), no data (X).

## Discussion

Our study reveals –for the first time– unevenness of ventilation distribution (elevated LCI) in children who were born with a CTM and underwent lobectomy in early life. Ventilation inhomogeneities are of clinical relevance as they impair gas exchange efficiency and can detrimentally affect the distribution of inhaled medications [26]. Notably unevenness of ventilation distribution was also greater in those children who showed poorer outcomes in somatic growth. We also observed increased reactance in children with CTM suggesting abnormal elastic recoil, a pattern observed in lung fibrosis and potentially associated with peripheral airway obstruction and small lungs [27].

Only a few studies have been published so far focusing on long-term lung function outcomes in children who had surgery for CTM in early life and they employed spirometry and whole body plethysmography to determine airflow limitation and lung volumes. For instance, Naito et al. [28] performed spirometry, lung volumes and exercise testing in 28 children who underwent lobectomy at mean age of 13 months and found no impairment in FEV_1_, FVC and total lung capacity (TLC) and exercise capacity. Beres et al. [10] followed-up 15 children who had undergone surgery for suspected CTM in infancy. They concluded that children undergoing lung resection in infancy have normal pulmonary function tests with almost complete recovery post-surgery due to compensatory lung growth except for those with asthma who were found to have lower FEV_1_. Caussade et al. [29] performed spirometry in 8 patients who underwent lung surgery at mean age of 7.5 years for congenital as well as acquired pulmonary conditions and described normal FVC values. Postpneumonectomy compensatory lung growth occurs in children but it is unclear whether it recapitulates normal lung growth. A longitudinal follow-up study by Nakajima et al. [30] on 27 operated children with CTM found decreased FVC and increased RV/TLC in the early post- operative period, which recovered within 2 years, leading to the conclusion that alveolar multiplication rather than distension was responsible for the compensatory lung growth in the long term. The lack of healthy control groups however is a limitation in these studies.

We have conducted the most comprehensive lung function studies yet in children with CTM and have compared them to a healthy control group employing identical methodology. While our study also found no difference in FVC values, children with CTM show impaired FEV_1_ values but only those with asthma as described previously by Beres et al. [10]. Thus impaired FEV_1_ values likely represent medium to large airways disease involvement due to asthma in CTM children.

TLC in CTM children was above 100% predicted in 7 out of 8 measured (Table 3), These findings suggest that compensatory growth has allowed, in terms of lung volume, full recovery from lobectomy in early life and excludes restrictive lung disease as a consequence of CTM and/or lung surgery. Higher LCI and reactance in CTM patients may be observed independent of asthma and may be related to dysanaptic lung growth after lobectomy. Alternatively, abnormal lung growth may be a previously unrecognised feature of CTM. This latter hypothesis is difficult to test as the radiologically normal appearing lung surrounding the CTM lesion cannot be investigated functionally in isolation in non-operated CTM children.

The elevated LCI found in children with CTM demonstrates a strong inverse correlation between LCI and age-adjusted height. It is therefore possible that ventilation inhomogeneities in children with CTM - indicated by the higher LCI score- may be causally associated with poorer outcomes in somatic growth. However longitudinal studies starting in early life are required to confirm this speculation because the association between LCI and somatic growth may be a surrogate marker for other adverse health effects (e.g. lung infections) manifesting more commonly in CTM patients. A previous study compared somatic growth of children operated in infancy versus those operated in childhood, arriving at a conclusion that children with earlier surgery had poorer outcomes in terms of height (and weight) as compared to the ones operated in childhood [31]. However lung function was not determined in that study. Our results suggest that unevenness of ventilation distribution as indicated by elevated LCI is closely related to poorer outcomes in somatic growth. It has yet to be determined however whether a close association between LCI and somatic growth can also be found in other disease groups where LCI has emerged as a highly sensitive, age-independent measure of airway dysfunction [32-36]. Further studies are required to determine whether increases in LCI are prevented by optimal nutritional intervention or whether they directly result in poorer outcomes in somatic growth.

Our study has several limitations. Firstly, all children with CTM underwent surgical resection in early life precluding us from investigating the effects of this intervention (as compared to non-surgical management) on lung function outcomes. While these studies are ongoing, we believe it is unlikely that LCI and reactance will be unaffected by a large non-resected CTM -even if asymptomatic- thereby reducing any possible difference in those parameters between operated and non-operated children with CTM. Secondly, our cohort of healthy children is slightly younger than the CTM cohort. However, given the relationship between height and LCI in early childhood (LCI decreases with increasing height before the age of six years), this difference in age may have resulted –if anything– in an underestimation of the difference between the groups. Lastly, as expected for a rare disease our case numbers are low and our study was powered to only observe a large effect size and unable to detect smaller differences in other lung function parameters. Previous studies found a difference in LCI of one standard deviation between healthy and asthmatic children (and children with multiple trigger wheeze) [37]. The elevated LCI found in children with CTM demonstrates a strong inverse correlation between LCI and age-adjusted height. Although a pilot study we suggest ventilation inhomogeneities in children with CTM, indicated by the higher LCI score is associated with poorer outcomes in somatic growth.

Based on these findings we suggest that nitrogen MBW and IOS should be considered as sensitive tools to measure peripheral lung function in children with CTM. Additionally both these tests have strong feasibility in preschool children. Our study reveals –for the first time– residual ventilation inhomogeneities in children with CTM who underwent lung resection in early life which was most pronounced in those with poorer outcomes in somatic growth. The strong inverse correlation between LCI (but not any other lung function parameter) and age-adjusted height in children with CTM but not healthy children suggests an unexpected association between ventilation inhomogeneities which requires further investigations in other chronic lung disease.

## Conclusions

This study suggests that the measures of peripheral airway function are abnormal in children with CTM, even though the lesion was removed surgically in early life. The study population is small and further studies are required to gather more evidence. The functional impact of the statistically significant differences in pulmonary function also need to be assessed by future studies to determine if these differences are clinically relevant.

## Additional file

Additional file 1: Table S1. ISAAC questionnaire comparing the respiratory health of CTM v healthy children. **Table S2.** Details on the lung function of the healthy cohort of children.

## Abbreviations

CCAM: Congenital cystic adenomatoid malformation
CPAM: Congenital pulmonary airway malformation
CTM: Congenital thoracic malformation
FEV1: Forced expiratory volume in 1 second
FVC: Forced vital capacity
IOS: Impulse oscillometry
LCI: Lung clearance Index
MBW: Multiple breath washout
R5Hz: Resistance at 5 hertz
X5Hz: Reactance at 5 hertz
TLC: Total lung capacity
