# Supplementary material for: Ventilation inhomogeneities in children with congenital thoracic malformations
Source: BMC Pulm Med. 2015 Mar 25;15:25. doi: 10.1186/s12890-015-0023-1 (PMC4417263; doi:10.1186/s12890-015-0023-1)
Supplement: Additional file 1: Table S1. — ISAAC questionnaire comparing the respiratory health of CTM v healthy children. Table S2. Details on the lung function of the healthy cohort of children. [file 12890_2015_23_MOESM1_ESM.docx]

**Table S1: ISAAC questionnaire comparing the respiratory health of CTM v healthy children**

| **ISAAC questionnaire** | **CTM**  **n/N (%)** | **Healthy**  **n/N (%)** | **P value** |
| --- | --- | --- | --- |
| Has your child ever had wheezing or whistling in the chest at any time in the past? | 7/10 (70%) | 4/17 (24%) | **0.04** |
| Has your child ever had asthma? | 4/10 (40%) | 0/17 (0%) | **0.01** |
| Does your child cough with a cold? | 10/10 (100%) | 15/17 (88%) | 0.52 |
| Does your child have a cough even without having a cold? | 3/10 (30%) | 4/17 (24%) | 1.00 |
| Has your child ever had Pneumonia?  Once:  More than once: | 1/10 (10%)  0/10 (0%)  1/10 (10%) | 0/17 (0%) | 0.37  N/A  0.37 |
| Has your child ever had Whooping cough?  Once:  More than once: | 0/10 (0%) | 1/17 (6%)  0/17 (0%)  1/17 (6%) | 1.00  N/A  1.00 |
| Has your child ever had Bronchiolitis?  Once:  More than once: | 3/10 (30%)  0/10 (0%)  3/10 (30%) | 1/17 (6%)  0/10 (0%)  1/17 (6%) | 0.13  N/A  0.13 |
| Has your child ever had Croup?  Once:  More than once: | 3/10 (30%)  1/10 (10%)  2/10 (20%) | 4/17 (24%)  1/17 (6%)  3/17 (18%) | 1.00  1.00  1.00 |
| Has your child ever had a problem with sneezing, or a runny or blocked nose when he/she DID NOT have a cold or flu? | 4/10 (40%) | 5/17 (29%) | 0.68 |
| In the past 12 months had any of the following medications to manage cough, wheeze or asthma?  Salbutamol, Ventolin, Asmol:  Flixotide, Seretide, Pulmicort:  Oral Steroids: | 4/10 (40%)  2/10 (20%)  1/10 (10%) | 4/17 (24%)  1/17 (6%)  3/17 (18%) | 0.41  0.54  1.00 |
| Has your child ever had eczema? | 4/10 (40%) | 4/17 (24%) | 0.41 |
| **Interview** | | | |
| Does your child have a doctors diagnosis of asthma? | 4/10 (40%) | 0/17 (0%) | **0.01** |

**answered yes to question (n)**

**Total number of children in group (N)**

**Table S2: Details on the lung function of the healthy cohort of children**

| **Age/sex** | **Height Z score** | **LCI** | **FEV_1_ % pred** | **FVC % pred** | **R5Hz Z score** | **X5Hz score** |
| --- | --- | --- | --- | --- | --- | --- |
| 5.4/F | -1.1 | 7.5 | b | 119 | 0.45 | -0.59 |
| 5.1/F | 0.1 | 8.7 | 86 | 92 | -0.86 | 0.39 |
| 4.5/M | 1.7 | 8.0 | 103 | 92 | 1.87 | -0.15 |
| 4.2/F | 1.1 | 6.9 | 118 | 127 | 0.87 | -1.43 |
| 5.0/M | 0.5 | 7.2 | 77 | 73 | 2.80 | -2.22 |
| 5.1/M | 0.6 | 7.4 | 128 | 131 | -0.39 | 0.04 |
| 5.0/M | 1.0 | 7.1 | 118 | 118 | 1.03 | 0.99 |
| 5.2/F | -0.1 | 6.5 | 116 | 110 | 1.70 | 0.62 |
| 4.7/F | 1.7 | 7.9 | 99 | 97 | 0.08 | -0.04 |
| 5.2/F | -0.8 | 7.2 | 109 | 129 | -1.02 | 0.30 |
| 4.7/F | 0.5 | 8.2 | 87 | 83 | 0.73 | 0.12 |
| 4.4/F | 0.2 | 6.8 | 127 | 119 | 0.77 | -0.15 |
| 4.0/F | 1.0 | 7.3 | 122 | 115 | 0.59 | 0.17 |
| 5.3/F | 0.3 | 6.6 | 146 | 141 | -1.55 | -0.09 |
| 4.6/F | 1.2 | 7.0 | 115 | 100 | -0.65 | 1.16 |
| 4.8/M | 0.8 | 7.7 | N/A | N/A | 0.59 | -1.22 |
| 4.3/F | **-**1.1 | 7.1 | 101 | 109 | 1.62 | 0.3 |
